# Vibration Monitoring of Civil Engineering Structures Using Contactless Vision-Based Low-Cost IATS Prototype

**DOI:** 10.3390/s21237952

**Published:** 2021-11-28

**Authors:** Rinaldo Paar, Ante Marendić, Ivan Jakopec, Igor Grgac

**Affiliations:** 1 Department of Applied Geodesy, Faculty of Geodesy, University of Zagreb, Kačićeva 26, 10000 Zagreb, Croatia; ante.marendic@geof.unizg.hr (A.M.); ivan.jakopec@geof.unizg.hr (I.J.); 2 PNT Tech d.o.o., Bukovački obronak 26, 10000 Zagreb, Croatia; igrgac.pnttech@gmail.com

**Keywords:** IATS prototype, accelerometer, bridge, load test, dynamic displacement and deformation monitoring, natural frequency

## Abstract

The role and importance of geodesists in the planning and building of civil engineering constructions are well known. However, the importance and benefits of collected data during maintenance in exploitation have arisen in the last thirty years due primarily to the development of Global Positioning Systems (GPS) and Global Navigation Satellite System (GNSS) instruments, sensors and systems, which can receive signals from multiple GPS systems. In the last fifteen years, the development of Terrestrial Laser Scanners (TLS) and Image-Assisted Total Stations (IATS) has enabled much wider integration of these types of geodetic instruments with their sensors into monitoring systems for the displacement and deformation monitoring of structures, as well as for regular structure inspections. While GNSS sensors have certain limitations regarding their accuracy, their suitability in monitoring systems, and the need for a clean horizon, IATS do not have these limitations. The latest development of Total Stations (TS) called IATS is a theodolite that consists of a Robotic Total Station (RTS) with integrated image sensors. Today, IATS can be used for structural and geo-monitoring, i.e., for the determination of static and dynamic displacements and deformations, as well as for the determination of civil engineering structures’ natural frequencies. In this way, IATS can provide essential information about the current condition of structures. However, like all instruments and sensors, they have their advantages and disadvantages. IATS’s biggest advantage is their high level of accuracy and precision and the fact that they do not need to be set up on the structure, while their biggest disadvantage is that they are expensive. In this paper, the developed low-cost IATS prototype, which consists of an RTS Leica TPS1201 instrument and GoPro Hero5 camera, is presented. At first, the IATS prototype was tested in the laboratory where simulated dynamic displacements were determined. After the experiment, the IATS prototype was used in the field for the purpose of static and dynamic load testing of the railway bridge Kloštar, after its reconstruction according to HRN ISO NORM U.M1.046—Testing of bridges by load test. In this article, the determination of bridge dynamic displacements and results of the computation of natural frequencies using FFT from the measurement data obtained by means of IATS are presented. During the load testing of the bridge, the frequencies were also determined by accelerometers, and these data were used as a reference for the assessment of IATS accuracy and suitability for dynamic testing. From the conducted measurements, we successfully determined natural bridge frequencies as they match the results gained by accelerometers.

## 1. Introduction

The process of acquiring data from experimental tests is inevitably influenced by the available technologies with their advantages and disadvantages. The common approach in data acquisition in civil engineering is based on contact point sensors (could be displacement, strain, velocity, or acceleration sensors) whose measurements are transferred via wired connections to the data acquisition hardware, which is rather complex, expensive, and time-consuming to set up. The elimination of physical installations of sensors on different structures is very attractive, especially for structures that might not be easily or safely accessible. Among contactless technologies, vision-based vibration monitoring is possibly the solution that attracts a lot of the interest on the part of civil engineers, given that the advantages of contactless monitoring can be potentially obtained through simple and low-cost instruments [1]. There are alternative technologies to avoid the use of connection cables (given the difficulties and efforts in cabling large structures) with wireless sensors [2,3]. In this article, the focus is on contactless vision-based vibration monitoring using a low-cost geodetic IATS prototype.

The monitoring of civil engineering structures or parts of the Earth’s surface involves periodic or continuous observations to estimate the object’s general current state, as well as the determination of the need for structure remediation, reconstruction, or destruction. The process involves the performance of different kinds of measurements using different sensors. The measurements and results must be precise and reliable, i.e., accurate, and tested for significance [4]. The measurement results represent an important parameter in assessing the condition and safety of the structures, and it is especially important for structures used beyond their designed lifetime. Any kind of damage or significant deformation affects the safety of the constructions, e.g., bridges, dams, towers, or skyscrapers, and this can result in their closure or even collapse [5]. The role and importance of geodesists in the planning and building of civil engineering constructions are well known, but the monitoring of artificial or natural structures is one of the key tasks in engineering geodesy, next to site surveying and setting out, which have a big role in planning and building. Geodetic monitoring is one aspect of monitoring systems in general. There are two subtypes of geodetic monitoring [4]:

- **Structural monitoring** (vibration-based) refers to the measurement and evaluation of dynamic displacements and natural frequencies of civil engineering structures such as bridges, tunnels, dams, railways, towers, or skyscrapers, i.e., generally manmade objects.
- **Geo-monitoring**, in contrast, is used as a term for the determination of changes, movements, or deformation of natural structures, such as landslides and slopes.

The main aim of geodetic monitoring is to determine statistically significant geometric changes in size, shape, and position between two or more measuring epochs [6]. According to the monitoring data, an action can be taken on the construction to prevent material and non-material damages. In the last thirty years, due to the development of Global Positioning Systems (GPS) and Global Navigation Satellite System (GNSS) instruments and sensors, which can receive signals from multiple GPS systems, structural monitoring has recently become common for geodesists. Any changes in the designed natural frequencies can be a sign of structural damage and a cause for alarm. Dynamic displacements and natural frequencies of objects can be determined by various types of sensors and instruments. Until fifteen years ago, geodetic instruments and sensors were only capable of displacement monitoring, while other sensors, namely accelerometers, had to be used for the determination of natural frequencies [7]. Today, modern geodetic instruments and sensors such as GNSS, RTS, and IATS can be used for static and dynamic bridge monitoring [8], being the main part of bridge load testing after its construction, reconstruction, or remediation.

Until the development of IATS, RTS and GNSS instruments were used for the purpose of geodetic monitoring. The limitation of the first RTS models was their instrument measurement frequency of 1 Hz, which was lower than the fundamental frequency of the bridges, as demonstrated in [9]. As RTS models have been developing, they could achieve measuring frequencies of 5–7 Hz. RTS instruments were used to measure simulated dynamic displacements for the purpose of analyzing the accuracy of dynamic measurements by RTS, as well as for the determination of dynamic displacements and natural frequencies of bridges in exploitation [10,11,12,13,14]. A state-of-the-art RTS instrument with 20 Hz measuring frequency for recording the changing of the 3D coordinate accuracy of a moving target was tested in [15]. The measurement of dynamic displacements and natural frequencies of the railway bridge Sava (Zagreb, Croatia) during the load testing after bridge reconstruction proved that RTS can determine dynamic displacements of the bridge in vertical and lateral directions, and the natural frequencies [16]. How to increase the measurement frequency of an RTS instrument from 7–10 Hz to 20 Hz and thus overcome the limitations of RTS regarding measurement frequencies is presented in [8].

GNSS sensors, systems, and instruments do not have such limitations regarding measuring frequencies. Today, GNSS receivers can achieve a measuring frequency of up to 100 Hz, but they still lack the same level of precision and accuracy in the horizontal and especially in the vertical direction, unlike RTS and IATS instruments. Consequently, the determination of dynamic displacements of rigid structures characterized by smaller values of dynamic displacements on a sub-centimeter level is limited due to the achievable measurement accuracy. However, regardless of this, they have become widely used to monitor dynamic displacements of large and flexible structures such as long bridges, towers, and skyscrapers, which are all characterized by large displacements (10 cm or more) and lower natural frequencies (less than 1 Hz). The results of GNSS monitoring of dynamic displacements of large-scale structures were shown in [10,17,18,19,20], where dynamic displacements, i.e., natural frequencies, of structures were successfully determined from GNSS measurements and the achieved results. At the moment, some studies are focusing on applying GNSS instruments for monitoring the dynamic displacement of more rigid structures with dynamic displacements on a millimeter level and higher frequencies [11], as well as on improving the GNSS measurement accuracy due to the limitations of ephemeris, multipath, and atmospheric errors and receiver measurement noise [21].

RTS with additional cameras integrated into the telescope are commonly denoted as Image-Assisted Total Stations (IATS) [22]. In the literature, a number of alternative terms exist for this class of instruments [4]: photo theodolite, video theodolite/tacheometer, image-assisted photogrammetric scanning station, and others. Due to the rapid technological development, these different sensor classes, each with their specific advantages, can be unified, utilized, and are fused as one single (nearly) universal geodetic instrument [23]. Modern TS are multi-sensor systems that can very precisely and accurately determine the three-dimensional coordinates of target points by combining horizontal angle, vertical angle, and distance measurements [8]. Today, many different sensors and measurement methods are combined in TS along with their integration into global navigation satellite system (GNSS) positioning, wireless communication and operation using a controller, additional cameras for documentation (Image-Assisted Total Stations—IATS), and IATS with a scanning function (Image-Assisted Scanning Total Stations—IASTS) [6].

Nowadays, IATS instruments can solve different engineering tasks and applications in semi-automated object reconstruction systems [24], fully automated deformation monitoring systems [25], industrial measurement systems [26], measurements of dynamic displacements by means of high-frequency image measurements [27], the capturing of additional information such as natural frequencies or intensity fluctuations of patterns using an image sensor to derive the temperature gradient of the atmosphere as a decisive influence parameter for angular refraction effects [28], and the monitoring of cracks [27,29]. IATS basic principles were shown and described in many studies [4,6,22,23,24,28,30,31,32,33]. In addition to the above-mentioned well known geodetic instruments, sensors, and systems, geodesists today can also perform structural monitoring of structures using state-of-the-art Terrestrial Laser Scanners (TLS) and ground-based radar interferometry. The procedure of measurements, the analysis of the acquired data, and the results of structural monitoring are explained and described in [34].

All instruments, sensors, and systems have advantages and disadvantages regardless of their application. One of the biggest disadvantages today is their high purchase price. In addition to offering high prices, manufacturers are still limiting export images and video resolution. Because of these facts, we have produced our own low-cost IATS prototype. This article focuses on the ability of the IATS prototype to measure dynamic displacements of bridges during excitation and its suitability to determine bridges’ natural frequencies from measured dynamic displacements. Determined frequencies are compared to theoretical results, obtained from the numerical model of the bridge, and to the results of the Operational Modal Analysis (OMA) based on acceleration measurements. Furthermore, this study aims to present, assess, and prove that the low-cost IATS prototype with an integrated image sensor in the form of a GoPro Hero5 camera is precise and accurate, i.e., reliable and suitable for the dynamic testing of civil engineering structures and for the determination of dynamic displacements and natural frequencies. In Section 2, the experimental study conducted in the laboratory is explained, as well as the application of the IATS prototype for the purpose of performing the load testing of the steel railway bridge Kloštar after its reconstruction in Vrbovsko, Croatia. The basic work and methodology for the determination of the dynamic displacements and natural frequencies from videos and images obtained with the IATS prototype are described as well. Following Section 2, the results and analysis are shown in Section 3 and the discussion is provided in Section 4, as well as the conclusion remarks and recommendations for future work.

## 2. Materials and Methods

In this paper, the focus is on the use of vision-based monitoring by a low-cost IATS prototype for low (from sub mm to cm level) and slow (<50 Hz) amplitude vibrations, as it is the case of most operational conditions in civil engineering. Even if the frame rate of the camera (acquired frames per second—fps) is high enough to avoid aliasing, effective algorithms (backed by adequate computational resources) as well as camera lens performances and quality are crucial to accurately track small structural motions.

Regarding video processing, the term optical flow [35,36] is used to identify general computer vision techniques associating pixels in a reference image with corresponding pixels in another image of the same scene. Among available algorithms, the most popular in structural engineering applications [37,38] appears to be correlation-based template matching and feature point matching.

For the purpose of bridge load testing, we have developed an IATS prototype that is explained in Section 2.1. Prior to load testing in the field, experimental testing was performed in the laboratory to see if the determination of dynamic displacements and natural frequencies is possible in ideal controlled laboratory conditions (Section 2.2). After it had been confirmed that it was possible, the testing of the prototype was conducted in the field during the load testing of the steel railway bridge Kloštar (Section 2.3). The IATS prototype measurement workflow concept and data processing are elaborated in Section 2.1.

### 2.1. IATS Prototype and Data Processing

Since currently available state-of-the-art IATS instruments such as Leica TS50 and TS60, Trimble S9, SX10, SX12, or Topcon DS-200i were not at our disposal and considering the fact that while using these instruments we could export the videos and images only with a VGA resolution of 640 × 480 px, we decided to develop our own prototype. These resolutions are not good enough for distanced objects, since the measuring points are represented by a very small number of pixels on images, regardless of high-quality optics with 30× time (or more) telescope magnification. The practical application of this approach in determining the dynamic displacements and natural frequencies must be considered since we cannot set up the instrument in the field close to the structure because of various obstacles. To overcome such limitations, we decided to develop our own IATS prototype.

**Figure 1:**
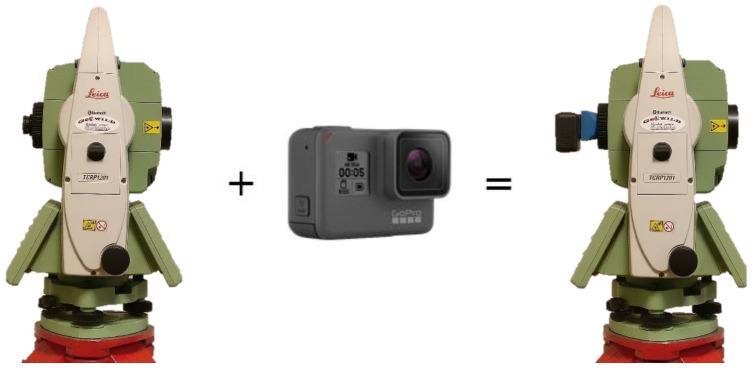
IATS prototype: Leica TPS1201 plus GoPro Hero5 camera.

The developed IATS prototype consists of a Leica TPS1201 instrument and GoPro Hero5 camera (Figure 1). Leica TPS1201 is a robotic total station with an angle measurement accuracy of 1” (ISO 17123-3) and distance measurement accuracy of 2 mm + 2 ppm (ISO 17123-4) [39]. The attached GoPro Hero5 camera on the ocular of the instrument telescope uses ultra-wide angle all-glass lens with reduced distortion. It offers a wide variety of different resolution modes for recording videos. A user can record videos from WVGA resolution at max 240 fps, 720 P resolution at max 240 fps, 960 P at max 120 fps, 1080 P at max 120 fps, 1440 P at max 80 fps, 2.7 K at max 60 fps to 4 K at max 30 fps. The user can choose from the different camera field of views (FOV) offered: narrow, linear, medium, wide, and superview [40]. The FOV and the video mode are directly correlated with the fps in a way that the wider the FOV and the higher the video resolution mode, the lower the fps that can be achieved. 

In order to fit the GoPro camera on the Leica TPS1201 ocular, an adapter was made by 3D printer. The adapter offers the possibility to directly attach the camera on the ocular of the Leica TPS1201 telescope. Before conducting the experiments, we performed the stability examination of the instrument telescope with the fitted GoPro camera in vertical direction. The test showed that there were no movements of the telescope because of its increased weight. The IATS prototype offers the possibility to operate the GoPro camera by smartphone application while we could operate the instrument via laptop computer. We thus do not have to touch the instrument or the camera, and we can ensure the stability of the instrument or horizontal sight of the telescope if necessary during the experiments.

**Figure 2:**
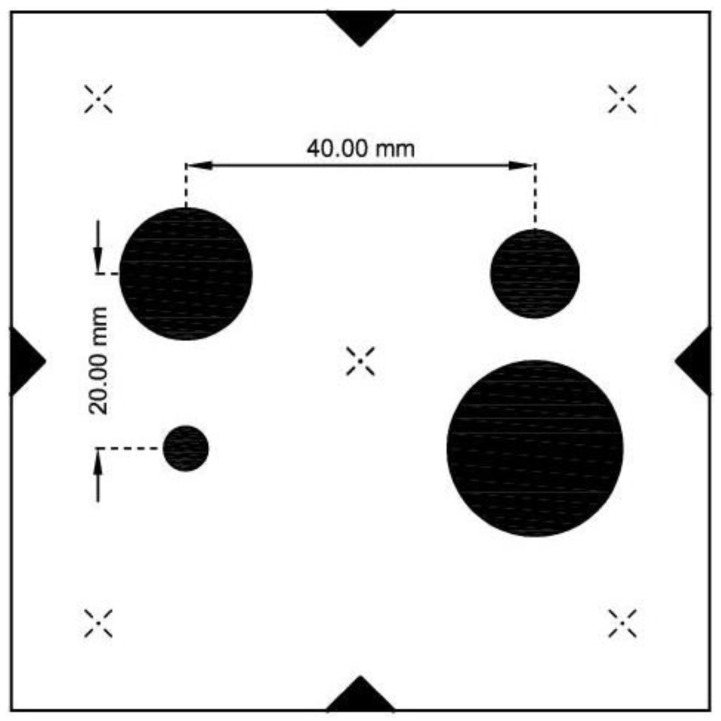
Photomark for target detection with predefined circle diameters and spacing between the circles’ centers.

Leica TPS1201 in the developed IATS prototype is used for the object target—photomark 30× magnification by instrument high-quality telescope optics and for GoPro Hero5 camera orientation in relation to the photomark on the object. During measurements the IATS prototype is placed away from the object on stable ground, which provides stability during the measurements. During the measurements, the camera records the video on the basis of which dynamic displacements and natural frequencies will be determined. In the course of data (video) processing, the image coordinates of photomark (Figure 2) in each epoch (frame) must be determined. The displacements in pixels are calculated as a difference between each epoch (frame) in relation to the reference epoch (frame). In the last step, the displacements are converted from image pixel units to linear units expressed in millimeters.

In order to process the video recorded by the camera, i.e., image processing to determine displacements and natural frequencies, we developed an algorithm in MATLAB. For image analysis, we used MATLAB image processing toolbox and for frequency spectra analysis we used MATLAB signal processing toolbox. For all other steps, we developed our own algorithm, which also contained the above-mentioned toolboxes. The main part of the analyzing strategy is the extraction of image coordinates of the center of the measurement photomark in each video frame and the comparison with the reference image. The predefined photomark for target detection between frames is shown in Figure 2. The photomark consists of four different circles with predefined diameters (5 mm, 10 mm, 15 mm, and 20 mm), as well as a predefined horizontal spacing between the circle centers of 40 mm and a vertical spacing of 20 mm, as shown in Figure 2.

**Figure 3:**
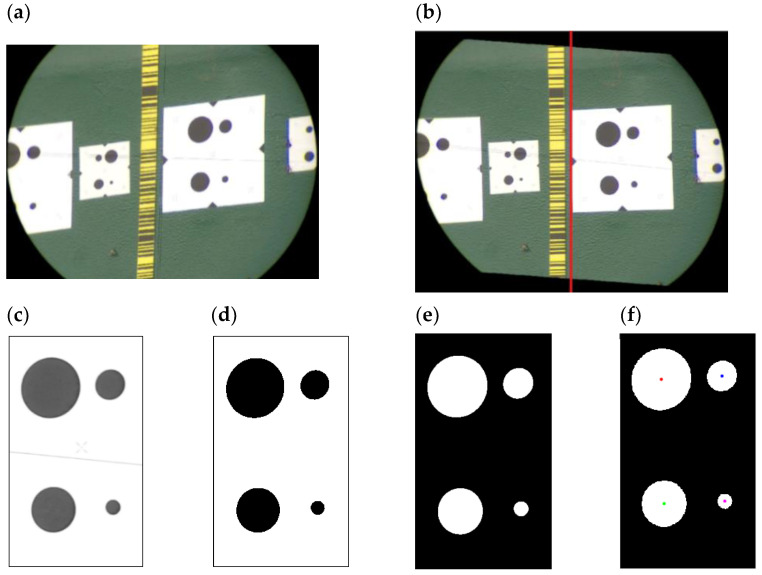
Representation of the photomark circle center detection workflow from video-extracted image frame (**a**), transformation to orthogonal projection (**b**), conversion to grayscale image (**c**), conversion to black and white image (**d**), inverting the black and white image (**e**), and circle detection (**f**).

To determine circle centers on the basis of the recorded video file, the video stream is split into single frames and subsequently analyzed. The developed algorithm performs several steps and is shown in Figure 3:

- First, the video frame is converted into single image frames, whereby each image represents one measuring epoch.
- Second, an image rectification to correct perspective-based distortion is performed. This effect is caused by a non-horizontal view of the IATS prototype telescope on the photomark signal and can be described and removed by a homography (projective transformation) based on the measured parameters: known distance, azimuth and zenith angle of the IATS prototype to the longitudinal axis of the bridge. The image is transformed into an orthogonal projection. In this phase, only the region around the photomark is defined in order to reduce the computation time.
- Third, the conversion from an RGB image to a grayscale image and then to binary black and white image is performed. In this way, the detection of circle boundaries (edge detection) and circle centers is easier.
- Fourth, inversion of black and white image is performed.
- Fifth, the circle center detection on images is performed by subpixel detection procedure of the circle’s centers.
- Sixth, based on the defined circle centers, the joint center of all four circles is calculated.
- Seventh, the differences of the calculated joint centers with respect to the first reference image can then be calculated, i.e., dynamic displacements. The dynamic displacements in time are calculated in the image coordinate system, i.e., in pixel units. Based on the known fps of the GoPro Hero5 camera, each frame is placed into the time domain of the recorded video frame and in relation to the first frame, i.e., reference frame dynamic displacements have been calculated.
- Eighth, as a function of the a priori known spacing between the circle centers, the scale factor is calculated and used to convert pixel displacements into millimeter displacements.
- Ninth, using Fast Fourier Transformation (FFT), the time domain is converted into the frequency domain, and the natural frequencies can then be calculated and determined.

Prior to the load testing of the bridge, we performed an experiment in the laboratory to prove that the IATS prototype can determine dynamic displacements with predefined amplitudes up to 1.0 mm (Section 2.2).

### 2.2. Experimental Testing in the Laboratory

**Figure 4:**
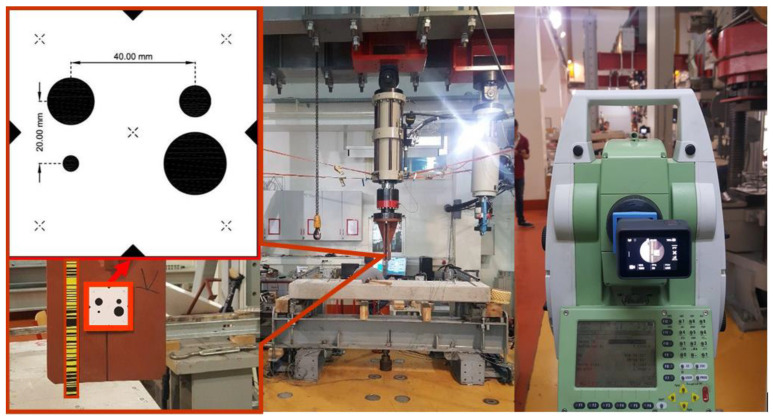
Photomark measuring signal (red on the **left**), testing machine (**middle**), and IATS prototype (**right**) setup in the laboratory for the purpose of measuring simulated dynamic vertical displacements with different amplitudes at 5.0 Hz frequency using multi-purpose universal testing machine.

Experimental testing was performed in the Structural Testing Laboratory of the Faculty of Civil Engineering, University of Zagreb prior to load testing. The tests were performed to see if the determination of dynamic vertical displacements and natural frequencies is possible in ideal controlled laboratory conditions. The dynamic vertical displacements were simulated by a multi-purpose universal testing machine intended for static and dynamic testing of mechanical properties of building materials and constructions and were measured by the IATS prototype, while a signal photomark was used for measuring with four predefined known circles’ radius and distance between the circles’ centers (Figure 4).

The test consisted of simulated displacements with the amplitudes of A = 0.2, 0.5, and 1.0 mm at the frequency of F = 5.0 Hz. We managed to determine all displacements and frequencies. Some of the similar previous experiments conducted in the laboratory are partially presented in [41]. In this section, we have presented the results of simulated displacements with different amplitudes at F = 5.0 Hz since the expected first natural frequency of the bridge was around F = 5.0 Hz and the expected dynamic displacement amplitude was A = 0.2 mm. In this way, we wanted to test and prove the ability of the IATS prototype to determine simulated frequency and dynamic displacements before performing the testing of the bridge in the field.

**Figure 5:**
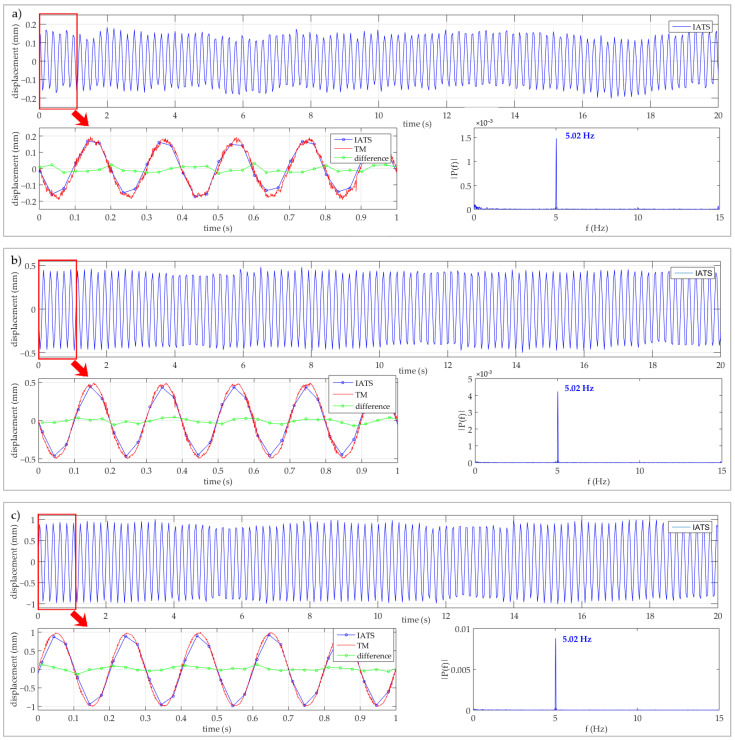
The measurements obtained by means of IATS prototype of simulated dynamic vertical displacements by TM, comparison of simulated and measured displacements with the difference between them and determined frequencies. In every test, the simulated predefined frequency was F = 5.0 Hz with different amplitudes of A = 0.2 mm (**a**), A = 0.5 mm (**b**), and A = 1.0 mm (**c**).

The IATS prototype was set up at the maximal distance from the testing machine that was possible in the laboratory, and it was d = 13.709 m. The IATS prototype GoPro Hero5 camera was set to narrow mode, 1080P with 30 fps for video recording. Measurements and results obtained by the IATS prototype and testing machine for all three experiments are shown separately in Figure 5a–c. For each experiment, first, (upper) Figure 5 shows 20 s of obtained measurements by the IATS prototype, second, (lower left) Figure 5 shows the comparison between simulated (TM—testing machine) and measured displacements (IATS prototype) with the difference between them for a time interval of 1 s, and third, (lower right) Figure 5 shows the determined frequency from IATS prototype measurements at predefined frequency F = 5.0 Hz with different amplitudes of A = 0.2 mm (a), A = 0.5 mm (b), and A = 1.0 mm (c).

Since the accuracy of the testing machine used to simulate dynamic displacements significantly exceeds the accuracy of the IATS prototype, the displacements and frequencies obtained by the testing machine were used as a reference. The first aim of the experiment was to test the ability of the IATS prototype to determine simulated dynamic vertical displacements lower than 1 mm. We can see in Figure 5 that in all three experiments, although the amplitudes of simulated vertical displacements were very low, they have been precisely determined by the IATS prototype. Since the displacements obtained by means of the testing machine were used as a reference, the differences between the displacements measured by the IATS prototype and by the TM represent errors of displacements determined by the IATS prototype. These errors were within the interval from Δ = −0.03 mm to 0.06 mm for A = 0.2 mm, from Δ = −0.11 mm to 0.08 mm for A = 0.5 mm, and from Δ = −0.15 mm to 0.13 mm for A = 1.0 mm (green line in lower left Figure 5a–c). Standard deviations of displacement errors in the experiments were σ = 0.02 mm, σ = 0.04 mm, and σ = 0.06 mm, which indicates a high level of measurement accuracy achieved by the IATS prototype.

The second aim of the experiment was to test the ability of the IATS prototype to determine the oscillation frequency of the simulated dynamic displacements with oscillation amplitudes of less than A = 1 mm and F = 5 Hz oscillation frequency. Dominant frequencies of oscillations in all tests were calculated using the Fast Fourier Transform (FFT) analysis. The results of determined frequencies from measurement data obtained by means of IATS prototype are shown in Figure 5a–c (lower right). Based on the comparison with simulated oscillating frequency, we can conclude that the IATS prototype was able to determine simulated frequency in all three tests with a high level of precision.

The constant difference in determined frequency of Δ = +0.02 Hz in all three tests is related to the fps not being achieved by the GoPro Hero5 camera as predefined. This occurs in every video recorded by the GoPro Hero5 camera because the achieved frame rate is not equal to the predefined rate, i.e., it can be smaller or higher from than the predefined rate. However, it is not relevant for the main purpose of the camera, but only for the purpose when time synchronizations are relevant.

Table 1 shows minimal, maximal, and mean oscillation amplitudes with their standard deviations and best fit sinusoidal amplitude determined by the IATS prototype and TM. The mean amplitudes in all experiments determined by IATS differ from the mean amplitudes determined by TM up to maximally 0.05 mm. Due to the IATS prototype measurement frequency of 30 Hz (GoPro Hero5 camera was set up to 30 fps for video recording), some amplitudes of the simulated displacements at F = 5.0 Hz were not determined equally as simulated because maximal amplitude was achieved between two IATS measurements (between two frames of recorded video). This can be noticed on the lower left part of Figure 5 (the differences between TM and IATS prototype are presented by the green line) and from Table 1 (differences between TM and IATS min and max values of determined amplitude). The calculated differences between TM and IATS with their standard deviations of 0.012 mm and 0.033 mm can be characterized as very precise. Furthermore, the IATS prototype sampling rate of 30 Hz does not influence the accuracy in determining the frequency of simulated vertical displacements, since according to the Nyquist theorem, the minimum sampling rate condition (two times higher) for determining the oscillation frequency is met. Nevertheless, we decided that for the measurements during the dynamic load testing of the steel railway bridge Kloštar, the GoPro Hero5 camera would be set up to record a movie at 60 fps, since the expected frequencies of the bridge were higher than during the experimental testing in the laboratory, and we also wanted to determine dynamic displacements with a high level of accuracy as much as possible, i.e., we wanted to record a more detailed response of the bridge during dynamic testing.

### 2.3. Case Study: Load Testing of the Steel Railway Bridge Kloštar

**Figure 6:**
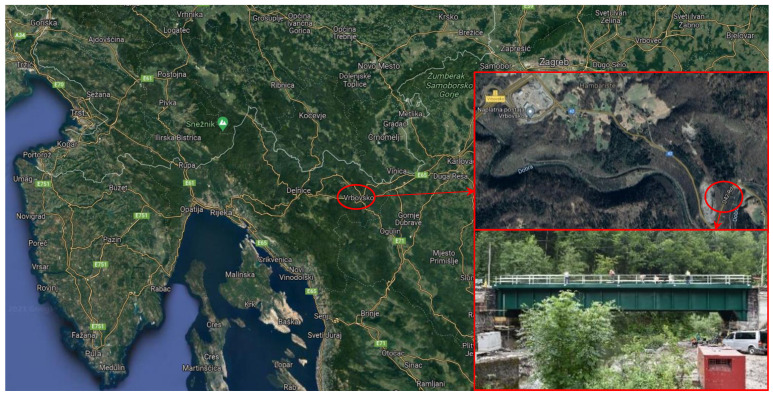
Location of the railway bridge Kloštar over the river Dobra near the city Vrbovsko in the central part of the Republic of Croatia and the bridge itself.

Detailed load testing of the steel railway bridge Kloštar over the river Dobra near the city Vrbovsko in the central part of the Republic of Croatia (Figure 6) was conducted after its reconstruction. The load testing was performed according to Croatian National Standard HRN U.M1.046, which requires load testing to be performed after the reconstruction is completed and prior to its opening for traffic. According to the Croatian legislation, every railway bridge with a span longer than 10 m must be tested before opening for traffic. The purpose of the load testing is to empirically quantify the load bearing capacity of the structure according to the project.

Load testing on structural materials is a common practice used to evaluate their mechanical characteristics and performances, e.g., strength and deformation capacity under static or dynamic load. Generally, the load testing of the bridge consists of static and dynamic testing [42]. In static load testing, RTS and GNSS instruments are mostly used depending on the characteristics of the tested bridge and the values of displacements expected from the model, precise levels. Older models of RTS and GNSS instruments were not used for dynamic testing in the past due to their limitations in sampling frequency and achievable precision. Due to constant improvements of RTS and GNSS instruments, they are no longer limited only to measuring the static displacements of the structures. Today, they are frequently used for the monitoring of dynamic displacements during their exploitation as a part of the monitoring system installed on the structure.

In the last fifteen years, GNSS instruments with sampling frequency 10–20 Hz have been generally used for monitoring the displacements of large and flexible bridges [43,44]. The major requirement for GNSS measurements to be conducted is a clean horizon. This cannot always be met during load testing because the passing trains or trucks deform or even disrupt the satellite signal [45,46]. So, the alternative was found with newer models of RTS, which can very precisely measure the position of the moving point (reflector) with a sampling frequency of up to 20 Hz [15]. RTS do not need a clean horizon; however, they require direct visibility between the reflector mounted on the moving point of the bridge and RTS with the distances between them up to few hundred meters. However, RTS can achieve submillimeter to millimeter levels of precision while recording 3D coordinates of a moving target, unlike GNSS, which can achieve a few millimeters to centimeter levels of precision [47]. There have been a few studies where RTS instruments with sampling frequency 5–7 Hz were used for measuring simulated and actual dynamic displacements of bridges [12,13,14,46,48]. Although modern RTS can achieve a sampling frequency of up to 20 Hz, this is very often not the case, and they can be found very rarely among different models and types provided by all manufacturers. However, due to the development of IATS that have cameras (image sensors) integrated into the RTS instruments for taking images and recording videos, the achievable sampling rate actually corresponds to the video frame rate, i.e., frames per second (fps), and responds to 30 fps or 30 Hz. Image sensors integrated into RTS are placed on and/or into the instrument telescope and thus use telescope optics with magnification up to 30×. With image and video processing, we can achieve a submillimeter level of precision while determining the displacement of a moving target. According to the Nyquist theorem, we can determine natural frequencies of structures two times smaller than the instrument measuring sampling rate, e.g., we can determine frequencies up to 15 Hz if we have an instrument with a sampling rate of 30 Hz for recording the vibration signal of the structure. In the case of deformation and vibration monitoring, this is the greatest advantage of current state-of-the art IATS instruments.

**Figure 7:**
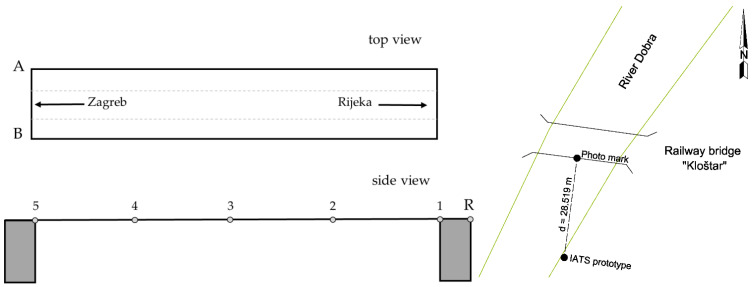
Schematic representation of the bridge (left). Top view shows the measuring lines (A, B) for geometric leveling of the bridge, while side view shows measuring spots (1–5) on measuring lines for the purpose of static load testing. The sketch on the right side represents IATS prototype setup in the field in relation to the bridge for the purpose of dynamic load testing.

For the purpose of steel railway bridge Kloštar load testing, i.e., for the static testing, we applied the geometric leveling method with two precise levels and parallel-plate micrometers since they offer a high level of precision (sub mm) and they are most appropriate for static load testing of the short span bridges with small vertical displacements (sub cm and sub mm). The levels used were Leica NA2 with GPM3 parallel-plate micrometer and invar leveling rods with achievable standard deviation per 1 km double-run leveling of 0.3 mm according to ISO norm 17123-2 Optics and optical instruments—Field procedures for testing geodetic and surveying instruments—Part 2: Levels [49]. During static testing, the precise levels were set up outside of the bridge between the reference point R and first point 1. Ten measuring points were stabilized on the bridge and two reference points outside the bridge in two lines A and B. For dynamic testing, we used the developed low-cost IATS prototype and accelerometers. The IATS prototype measured the modal parameters of the bridge in point 3. Since the bridge did not have any characteristic points, i.e., unsignalized targets suitable for image processing and target identification between frames, we used predefined photomarks (Figure 2) for target detection between the frames. The IATS prototype was placed at a distance of d = 28.519 m approximately perpendicular to the longitudinal axis of the bridge in the middle of the bridge span, point 3 (Figure 7), while the accelerometers were set up on the bridge in all measuring points. The atmospheric conditions, which always have an influence on all geodetic measurements and can be significant, were ideal during this load testing. The air temperature, pressure, and humidity were constant. Most importantly, the temperature was 14 °C, without direct sunlight, since the sky was moderately cloudy with white clouds. However, we must emphasize that direct sunlight can be a problem for this type of measurement with the IATS prototype, since we are performing measurements with a camera and this must be taken into account. If this occurs during measurements in the field, it is necessary to set up the IATS prototype so it is not directed into the sunlight because then the recorded movie and extracted images from it can be of poor quality.

By setting up the IATS prototype perpendicular to the bridge longitudinal axis, we achieved the optimal position for the determination of the bridge’s vertical displacements, because the longitudinal axis of the bridge is parallel to the image sensor of the IATS prototype [8,50]. The GoPro Hero5 camera was set up to record a movie with the following settings: narrow FOV, 1920 × 1080 px, at 60 fps. With the camera thus set up and by using the instrument’s telescope 30× magnification at a distance of 28.519 m, we achieved the resolution of 0.333 mm/pixel., i.e., the smallest circle with 5 mm in diameter on the photomark is represented with 15 pixels.

For static and dynamic testing, there was one train composition used as bridge load, consisting of a locomotive and 2 freight wagons. The mass of the locomotives was 80 t (4 axles, 20.0 t per axle). The wagons were loaded with gravel, and their average mass was 79.8 t (4 axles, 19.95 t per axle). Total mass per train composition was approximately 240 t. Static testing was conducted in four phases. The first phase of the testing was a bridge without the load, in the second and third phases the bridge was loaded with differently placed trains on the bridge, while in the fourth phase the bridge was no longer loaded. In such a way, we recorded the initial state of the bridge in the first phase, while in the second and third phases we recorded the state of the bridge under load to empirically quantify the load bearing capacity of the structure according to the project. During the second and third phases, vertical displacements in measuring points are expected as indicated by the bridge model. After that, in fourth phase, the load is removed from the bridge and it should return to its initial state with some allowed tolerances according to the bridge design and material. During dynamic testing, three train passages with different speeds over the bridge were recorded. The details of these train passages are shown in Table 2.

## 3. Results

In Section 3.1, Section 3.2 and Section 3.3, the results obtained in static and dynamic testing of the bridge are presented and elaborated.

### 3.1. Static Displacements Determined by Geometric Leveling

Table 3 shows the results of the static load testing of the bridge, i.e., vertical displacements of the bridge under load (phase 2 and 3) and without the load (phase 4). Table 3 shows the second, third, and fourth phase in relation to the first phase. The maximal vertical displacement is detected in the second phase in the middle of the bridge span in point 3, line A and its value is 8.8 mm, while in line B it corresponds to 8.1 mm. The vertical displacements are slightly smaller in the third phase (8.5 mm in line A and 7.8 mm in line B) because of the different positions of the train on the bridge. From the results obtained in the fourth phase, we can conclude that the bridge returned to its initial state recorded in the first phase with the maximal residual of vertical displacements being 0.2 mm, which is within the allowed tolerances for this type of bridge.

Slightly higher vertical displacements achieved in line A in phases 2 and 3 are the consequence of the transverse slope of the bridge from line B to line A, i.e., the bridge is not horizontal in the transverse cross section as it is sloped from line B to line A, which results in higher pressure of the load placed on the bridge during static load testing and results in higher vertical displacements in line A.

### 3.2. Dynamic Displacements Determined by IATS Prototype

**Figure 8:**
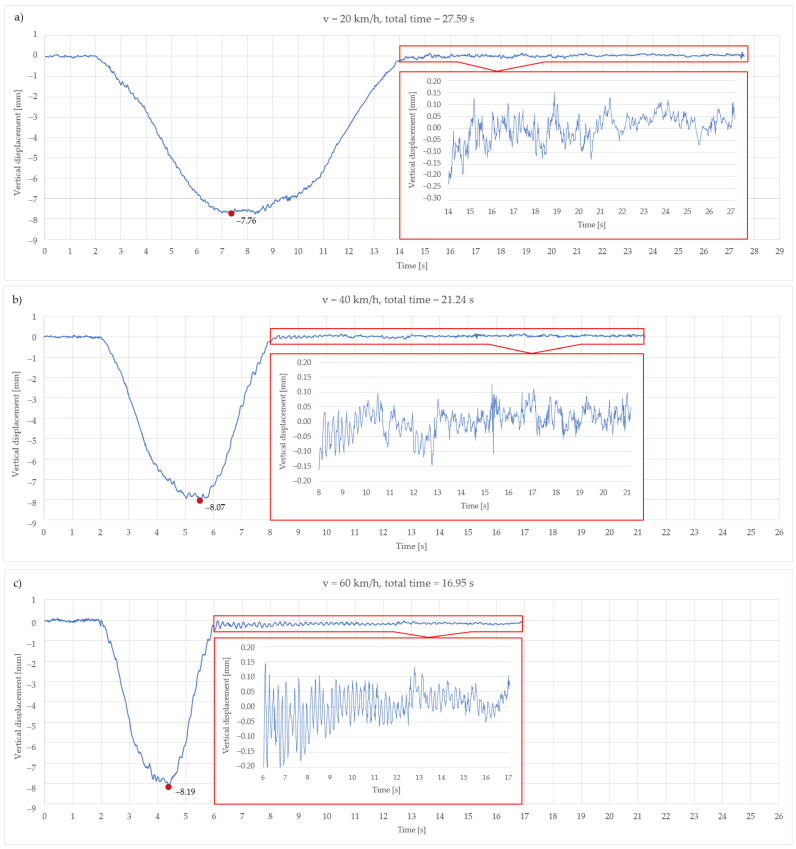
Determined vertical displacements obtained from IATS prototype raw measurement data—vertical displacements in the middle of the bridge span with marked maximum vertical displacement when the train is passing at 20 km/h (**a**), 40 km/h (**b**), and 60 km/h (**c**).

Figure 8 shows the determined vertical displacements obtained from IATS prototype raw measurement data, i.e., vertical displacements in the middle of the bridge span (point 3 on Figure 7 side view) with marked maximum vertical displacement during all train passages.

Every figure shows all measurement data recorded from the arrival of the train to the bridge to the departure of the train from the bridge. The departure of the train from the bridge differs between the passes because of the train speed. It is evident from Figure 8a that the train leaves the bridge after fourteen seconds while crossing the bridge at 20 km/h. After that, the next fourteen seconds represent the period in which the bridge is settling down. It can be noticed from the second to fourteenth second that the maximal determined vertical displacement is 7.76 mm, which corresponds to determined vertical displacement during static load testing in phase 3, line B, at measuring point 3 (Table 3).

It is evident from Figure 8b that the train leaves the bridge after eight seconds while crossing the bridge at 40 km/h. After that, the next thirteen seconds represent the period in which the bridge is settling down. It can be noticed from the second to eighth second that the maximal determined vertical displacement is 8.07 mm, which corresponds to determined vertical displacement during static load testing in phase 2, line B, at measuring point 3 (Table 3).

It is evident from Figure 8c that the train leaves the bridge after the sixth second while crossing the bridge at 60 km/h. After that, the next eleven seconds represent the period in which the bridge is settling down. It can be noticed from the second to sixth second that the maximal determined vertical displacement is 8.19 mm, which also corresponds to determined vertical displacement during static load testing in phase 2, line B, at measuring point 3 (Table 3).

From the results obtained with the IATS prototype during dynamic load testing after the train has left the bridge, it can be noticed that dynamic displacements were in the range of ± 0.15 to 0.20 mm. The results achieved by the IATS prototype related to determined vertical displacements during dynamic load testing while the train is on the bridge show a high level of coincidence with the results obtained by means of geometric leveling during static load testing.

In Figure 8a–c, the enlarged part (red rectangle) shows the dynamic oscillations of the bridge after the train has left the bridge, i.e., the settlement of the bridge after the train excitation, and this measurement part was used for the determination of the dominant natural frequencies of the bridge by the IATS prototype. Determined natural frequencies are presented in Section 3.3.

### 3.3. Analysis of Determined Natural Frequencies of the Bridge

**Figure 9:**
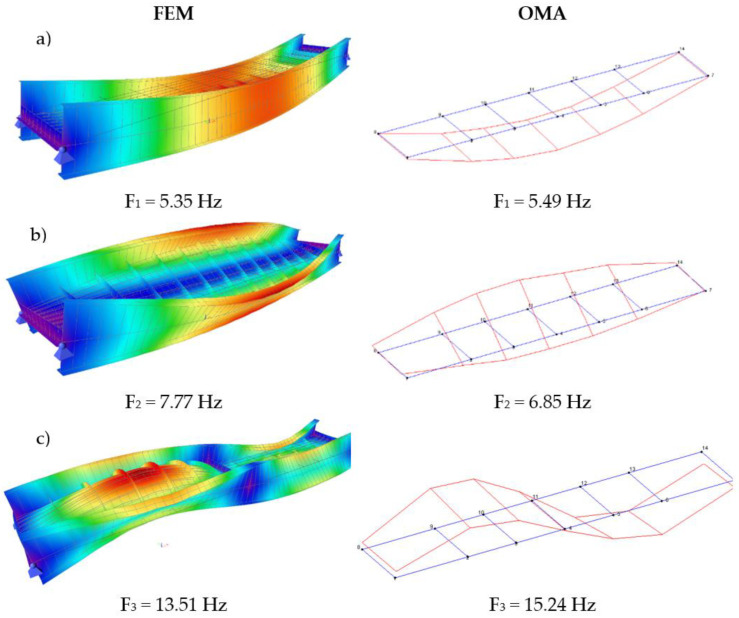
First (**a**), second (**b**), and third (**c**) modal shape and frequencies computed by FEM (left side on figures (**a**–**c**)) and determined by means of accelerometers during the dynamic load testing of the bridge (right side on figures (**a**–**c**)).

**Figure 10:**
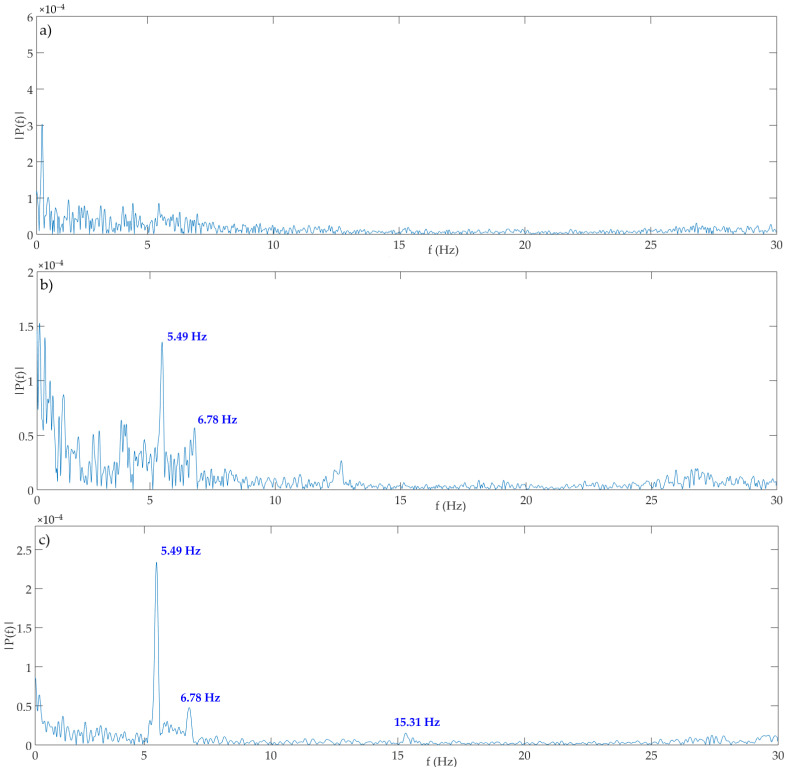
Amplitude spectrum of vertical component obtained from IATS prototype measurements: (**a**) excitation by train passing at 20 km/h—no frequency was detected; (**b**) excitation by train passing at 40 km/h—first and second frequency were detected; (**c**) excitation by train passing at 60 km/h—first, second, and third frequency were detected.

According to the achieved results related to the determination of oscillation frequencies during experimental testing in the laboratory, we wanted to determine the dominant natural frequencies of the bridge in the field as well. Dominant natural frequencies of oscillations in all dynamic tests were calculated using the Fast Fourier Transform (FFT) analysis. Computed modal shapes and frequencies by the Finite Element Method (FEM) as well as determined by accelerometers are shown in Figure 9. The results of frequencies determined from measurement data obtained by means of the IATS prototype are shown in Figure 10a–c and in Table 4. The aim of the testing was to determine the natural frequencies of the bridge during vibration monitoring with the IATS prototype. To assess the accuracy of our IATS prototype, we used ambient vibration measurements and the results of OMA based on acceleration measurements as a reference, since accelerometers with high-quality shielded cables are commonly adopted for data acquisitions during vibration monitoring [1]. Differences between the FEM and OMA based on acceleration measurements occur regularly because the FEM model is made by using design parameters that always differ from the constructed bridge. Therefore, natural frequencies determined with the IATS prototype are compared with frequencies obtained with OMA using accelerometers.

Figure 9 (left side) shows the computed first modal shape of the bridge at 5.35 Hz frequency, the second modal shape at 7.77 Hz frequency, and the third modal shape at 13.51 Hz frequency using the Finite Element Method (FEM). Figure 9 (right side) shows the first, second, and third modal shapes and frequencies obtained from ambient vibration measurements using accelerometers (OMA). The first modal shape of the bridge obtained by means of accelerometers was 5.49 Hz frequency, the second modal shape was 6.85 Hz frequency, and the third modal shape was 15.24 Hz frequency.

From Figure 10, which shows amplitude spectrums of vertical components obtained by means of IATS prototype measurements during excitation by trains passing at 20 km/h, 40 km/h, and 60 km/h, we can see the determined natural frequencies of the bridge. In Figure 10, the Y-axis represents amplitude, i.e., amplitude peaks expressed in meters ×10^−4^ (tenth of a millimeter), while the X-axis represents determined frequency in Hz.

Table 4 shows the natural frequencies determined with the IATS prototype for all train passages, as well as the frequencies computed by the FEM, and the comparison with the frequencies obtained by OMA based on acceleration measurements.

During the first excitation by the train passing at 20 km/h, we could not determine any frequency (Figure 10a, Table 4). The reason for that is that the excitation was not large enough, i.e., the bridge response to the excitation and dynamic vibrations (oscillations) were small and in the range of IATS prototype measurement accuracy.

During the second excitation by the train passing at 40 km/h, we determined the first two frequencies of the bridge (Figure 10b, Table 4). The first natural frequency determined by the IATS prototype matched the first natural frequency of the bridge measured by accelerometers, while the second natural frequency determined by the IATS prototype slightly differed from the one determined by accelerometers. At 40 km/h, the first frequency F1 = 5.49 Hz detected by the accelerometers was detected by the IATS prototype as F1 = 5.49 Hz. The second frequency F2 = 6.85 Hz detected by the accelerometers was detected by the IATS prototype as F2 = 6.78 Hz.

During the third excitation by the train passing at 60 km/h, we determined the first three frequencies of the bridge (Figure 10c, Table 4). The first natural frequency determined by the IATS prototype matched the first natural frequency of the bridge measured by accelerometers, while the second and third natural frequencies determined by the IATS prototype slightly differed from the one determined by accelerometers. At 60 km/h, the first frequency F1 = 5.49 Hz detected by the accelerometers was detected by the IATS prototype as F1 = 5.49 Hz. The second frequency F2 = 6.85 Hz detected by the accelerometers was detected by the IATS prototype as F2 = 6.78 Hz. The third frequency F3 = 15.24 Hz detected by the accelerometers was detected by the IATS prototype as F2 = 15.31 Hz.

## 4. Discussion

The presented study deals with the determination of dynamic displacements and natural frequencies, i.e., vibration monitoring in a controlled environment in the laboratory and in the field during load testing of the steel railway bridge Kloštar using a contactless vision-based technique of the developed low-cost IATS prototype and its comparison with conventional contact accelerometer sensors. The basic principle of the IATS prototype is elaborated with a focus on the analysis of data obtained from measurements.

The results from the experimental testing conducted in the laboratory (Section 2.2) showed that with IATS prototype sub mm displacements with amplitudes of A = 0.2 mm, 0.5 mm, and 1.0 mm at F = 5.0 Hz, the frequency can be determined by standard deviation of σ = 0.012 mm to 0.033 mm, i.e., sub mm displacements can be detected with a high level of accuracy and precision. The IATS prototype was placed at approximately 13 m from the TM. The displacement errors that occurred and the differences between simulated displacements by the TM and those detected by the IATS prototype are in a range from Δ = −0.15 mm to 0.13 mm depending on the simulated amplitude, but were detected with a high level of precision, i.e., with standard deviations of displacement errors of σ = 0.02 mm, σ = 0.04 mm, and σ = 0.06 mm. The simulated frequency by the TM of F = 5.00 Hz was detected by the IATS prototype in every test as F = 5.02 Hz.

After successfully determining simulated displacements and frequencies during the experiment in the laboratory, we tested the IATS prototype in the field during load testing of the steel railway bridge Kloštar. In Table 4, all determined natural frequencies are shown. Based on the comparison with natural frequencies obtained by means of OMA based on acceleration measurements, we can conclude that the IATS prototype was able to determine frequencies during the second and the third train passage. However, it must be noted that the possibility to determine natural frequencies largely depends on the amount of excitation, i.e., in our case it depended on the train speed, which directly correlates with the excitation. The larger the speed of the train, the larger the excitation and the bridge response to it, which means that dynamic vibrations are larger.

From Table 4, we can observe a very good correlation of the measurements achieved by the IATS prototype with those obtained by means of OMA based on acceleration measurements, and our findings can be considered very promising for the developed low-cost IATS prototype. The difference in the first determined frequency is below 0.01 Hz. In the second and third determined frequencies, the difference is below 0.10 Hz. The third determined frequency is determined only during the time when the train is passing at 60 km/h.

The major advantages of the developed low-cost IATS prototype and its potential in structural monitoring are presented in this study. The achieved precision and accuracy of measured displacements are at a high level because contactless vision-based measurements are performed by the GoPro camera, which uses 30× optical magnification of RTS Leica TPS1201. We have thus demonstrated that the IATS prototype position at approximately 30 m distance from the bridge ensures that one pixel represents 0.333 mm, which provides a high level of accuracy and precision in the determination of dynamic vertical displacements of a moving target (submillimeter level). The capacity of the camera to record video by 30 fps or 60 fps made it possible to determine natural frequencies of the railway steel bridge with a high level of accuracy and precision, i.e., the determined natural frequencies matched the frequencies determined by accelerometers with, on average, 99.51% overlap. Other advantages are related to the fact that the IATS prototype does not need to be placed on the bridge, and thus access to the monitored bridge is not necessary. In our case, we only had to put the photomark on the bridge since the bridge did not have any characteristic details for later data processing. The fact that our approach needs lesser time for field work and needed analysis is no less important. To set up the IATS prototype in the field, we needed three minutes in total. To put the photomarks on the bridge, we used self-adhesive paper, i.e., stickers, and we needed another two to three minutes. Image processing, i.e., video processing, does not require a lot of time, since the train passes were short and lasted thirty seconds max. We were recording full HD video, so when we were automatically extracting images from videos, we were extracting images with dimensions 1920 × 1080 px @ 30 fps, for the duration of 30 s. For the slowest train pass, we had approximately 900 images, which file size was not very big. The process to extract images from videos was performed in a couple of seconds. After that, we imported images into MATLAB for image processing and the determination of displacements and natural frequencies. For this purpose, we needed ten minutes. This process largely depends on bridge length, train length, and speed, the used video resolution, and available computers. However, from the conducted study, we can conclude that it is not time-consuming.

The price of the presented IATS prototype is much lower than that of a commercial IATS, which is a very important parameter to consider. The developed IATS prototype consists of a GoPro camera, which costs around EUR 500, and a Leica TPS1201 instrument from 2005, which costs around EUR 4000–5000. So, the prototype price is approximately EUR 5000, which in comparison with modern state-of-the-art IATS instruments is low since their price starts at around EUR 50,000 depending on the manufacturer, type, model, and additional equipment needed. We must also emphasize that we could place the GoPro camera, or some other available camera with similar characteristics, to any other new total station, which also costs around EUR 5000, since all available total stations on the market today offer high-quality optics with a 30× time (or more) magnification telescope. Contactless methods have distinct advantages over contact methods since they generally measure visible light, can be easily set up, and measure a large scene of interest as every pixel collects a time series. However, the tradeoff is less precise data compared to contact techniques [51]. Nevertheless, in our case, we managed to overcome this lack of precision by combining the video camera with the high-quality optics of the Leica TPS1201 robotic total station, which resulted in the developed IATS prototype. We can also conclude that our approach of combining the IATS prototype with accelerometers represents a data fusion method. Combining the contactless vision-based vibration monitoring performed by the low-cost IATS prototype with the data from accelerometers enables the denoising of measurements and provides better IATS prototype result estimates. This combination of the IATS prototype and accelerometers overcomes the field testing limitations of vision-based monitoring and has the potential for accurate and robust sensing on bridge structures, as previously presented only for cameras and accelerometers in the study [52].

## 5. Conclusions

The conducted study confirmed our aims and hypotheses regarding the potential of the developed IATS prototype, both in the laboratory and in the field during load testing of the steel railway bridge Kloštar. However, it also opened the future work that is needed for wider implementation of our approach. Future work is needed with regard to displacement measurement in more than one point signalized on the bridge when trying to achieve the same level of high measurement accuracy and precision. This approach will subsequently result in lower resolution and reduced quality of displacement measurements, since we will have to move away from the bridge at a longer distance so that we can accomplish a wider camera FOV. It is therefore important to test the camera with a higher video recording resolution (4K). It is also not easy to signalize and safely approach the points on the bridge in many projects, and in our future work we will test the IATS prototype when measuring displacements without signalized points on the bridge, i.e., we will use characteristic points on the bridge. The conducted study showed that our low-cost IATS prototype can be used for vibration monitoring, confirming the previous studies performed for commercial high-cost IATS [7,22,50]. In the end, it should also be highlighted that future technological development of commercial IATS instruments should include the integration of high-resolution and high frame rate (fps) image sensors into the telescope of the instruments, since this is not the case today. This study clearly shows that a high camera video resolution and high frame rate (fps) in combination with high-quality IATS telescope optics and its magnification provide very accurate and precise determination of dynamic displacements and natural frequencies of civil engineering structures.

## Figures and Tables

**Table 1:** Achieved min, max, and mean amplitudes with standard deviations and best fit sinusoidal amplitude for test F = 5 Hz, A = 0.2, 0.5, and 1.0 mm.

Test F = 5.0 Hz	A = 0.2 mm		A = 0.5 mm		A = 1.0 mm	
	TM	IATS	TM	IATS	TM	IATS
Min	0.178	0.134	0.484	0.436	0.980	0.885
Max	0.196	0.172	0.498	0.476	1.005	0.984
Mean	0.190	0.155	0.493	0.453	0.992	0.943
St.dev.		0.012		0.012		0.033
Best fit A		0.161		0.463		0.958

**Table 2:** Train passages during dynamic load testing.

Event	Excitation Type	Direction	Train Speed (km/h)
1	Passage of train	From east to west	20
2	Passage of train	From east to west	40
3	Passage of train	From east to west	60

**Table 3:** Static load test results of the steel railway bridge Kloštar over the river Dobra at 550 km + 630 m on the M202 Zagreb–Rijeka railway (mm).

	F2 (1)		F3 (1)		F4 (1)	
Measuring Point	Load—Train		Load—Train		Load—No Train	
	A	B	A	B	A	B
	(mm)	(mm)	(mm)	(mm)	(mm)	(mm)
1	0.2	0.3	0.3	0.3	0.0	0.0
2	6.9	6.4	6.6	6.1	0.2	0.2
3	8.8	8.1	8.5	7.8	0.2	0.2
4	6.8	6.6	6.9	6.4	0.1	0.2
5	0.2	0.2	0.3	0.2	0.0	0.0

**Table 4:** Computed natural frequencies using FEM, obtained by OMA and determined from IATS prototype measurements during bridge dynamic load testing.

	F_1_	F_2_	F_3_
FEM	5.35 Hz	7.77 Hz	13.51 Hz
OMA	5.49 Hz	6.85 Hz	15.24 Hz
	**20 km/h**		
IATS prototype	/	/	/
	**40 km/h**		
IATS prototype	5.49 Hz	6.78 Hz	/
	**60 km/h**		
IATS prototype	5.49 Hz	6.78 Hz	15.31 Hz

## Data Availability

The data presented in this study are available on request from the corresponding author. The data are not publicly available (they are part of the official load test of the Kloštar bridge, and as such are not publicly available).
